# Aqua­(2,9-dimethyl-1,10-phenanthroline-κ^2^ *N*,*N*′)bis­(3-hydroxy­benzoato-κ*O*)manganese(II)–2,9-dimethyl-1,10-phenanthroline–water (1/1/1)

**DOI:** 10.1107/S1600536809025926

**Published:** 2009-07-11

**Authors:** Xuejun Liu, Linyu Jin

**Affiliations:** a College of Chemistry and Chemical Engineering, Henan University, Kaifeng 475001, People’s Republic of China

## Abstract

In the title compound, [Mn(C_7_H_5_O_3_)_2_(C_14_H_12_N_2_)(H_2_O)]·C_14_H_12_N_2_·H_2_O, the Mn^II^ ion is coordinated by a bidentate 2,9-dimethyl-1,10-phenanthroline (dmphen) ligand, two monodentate 3-hydroxy­benzoate anions (3-HBA) and one water mol­ecule in a distorted trigonal-bipyramidal environment. An uncoordinated dmphen and an uncoordinated water mol­ecule cocrystallized with each complex mol­ecule. Intra- and inter­molecular O—H⋯N and O—H⋯O hydrogen bonds are also present between the coordinated 3-HBA and water mol­ecules and the uncoordinated dmphen and water mol­ecules in the crystal. The packing of the structure is further stabilized by π–π stacking inter­actions involving dmphen mol­ecules, with a centroid–centroid separation of 3.705 (3) Å.

## Related literature

For related structures, see Wang *et al.* (2003 ▶); Xuan *et al.* (2007 ▶); Xuan & Zhao (2007 ▶); Zhao *et al.* (2007 ▶, 2009 ▶). For bond-length data, see: Su & Xu (2005 ▶).
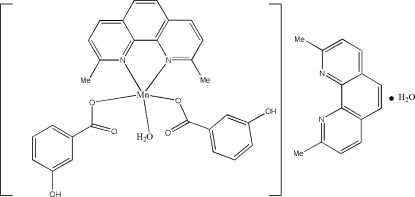


## Experimental

### 

#### Crystal data

[Mn(C_7_H_5_O_3_)_2_(C_14_H_12_N_2_)(H_2_O)]·C_14_H_12_N_2_·H_2_O
*M* *_r_* = 781.70
Monoclinic, 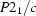

*a* = 14.7103 (16) Å
*b* = 18.578 (2) Å
*c* = 14.4598 (16) Å
β = 106.302 (1)°
*V* = 3792.9 (7) Å^3^
*Z* = 4
Mo *K*α radiation
μ = 0.41 mm^−1^
*T* = 296 K
0.37 × 0.35 × 0.12 mm

#### Data collection

Bruker SMART CCD area-detector diffractometer
Absorption correction: multi-scan (*SADABS*; Bruker, 1997 ▶) *T*_min_ = 0.864, *T*_max_ = 0.953
22827 measured reflections
7009 independent reflections
4112 reflections with *I* > 2σ(*I*)
*R*_int_ = 0.063

#### Refinement

*R*[*F* ^2^ > 2σ(*F* ^2^)] = 0.054
*wR*(*F* ^2^) = 0.151
*S* = 1.01
7009 reflections
502 parameters
H-atom parameters constrained
Δρ_max_ = 0.92 e Å^−3^
Δρ_min_ = −0.28 e Å^−3^

### 

Data collection: *SMART* (Bruker, 1997 ▶); cell refinement: *SAINT* (Bruker, 1997 ▶); data reduction: *SAINT*; program(s) used to solve structure: *SHELXS97* (Sheldrick, 2008 ▶); program(s) used to refine structure: *SHELXL97* (Sheldrick, 2008 ▶); molecular graphics: *SHELXTL* (Sheldrick,2008 ▶); software used to prepare material for publication: *publCIF* (Westrip, 2009 ▶).

## Supplementary Material

Crystal structure: contains datablocks I, global. DOI: 10.1107/S1600536809025926/pv2157sup1.cif

Structure factors: contains datablocks I. DOI: 10.1107/S1600536809025926/pv2157Isup2.hkl

Additional supplementary materials:  crystallographic information; 3D view; checkCIF report

## Figures and Tables

**Table 1:** Hydrogen-bond geometry (Å, °)

*D*—H⋯*A*	*D*—H	H⋯*A*	*D*⋯*A*	*D*—H⋯*A*
O8—H8⋯O1^i^	0.82	1.83	2.653 (4)	176
O5—H5⋯O7^ii^	0.82	1.88	2.686 (4)	168
O2—H4*W*⋯N3^iii^	0.83	1.96	2.764 (4)	162
O2—H3*W*⋯O3	0.83	1.80	2.617 (3)	165
O1—H2*W*⋯N4	0.83	2.15	2.951 (4)	161
O1—H1*W*⋯O3^iv^	0.85	1.99	2.839 (4)	180

Symmetry codes: (i) 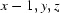; (ii) 
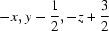
; (iii) 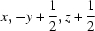; (iv) 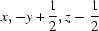.

**Fig. 1.**
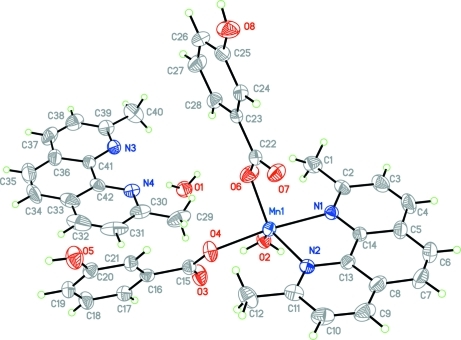
The molecular structure of (I), with atom labels and 30% probability displacement ellipsoids for Non-H atoms.

**Fig. 2.**
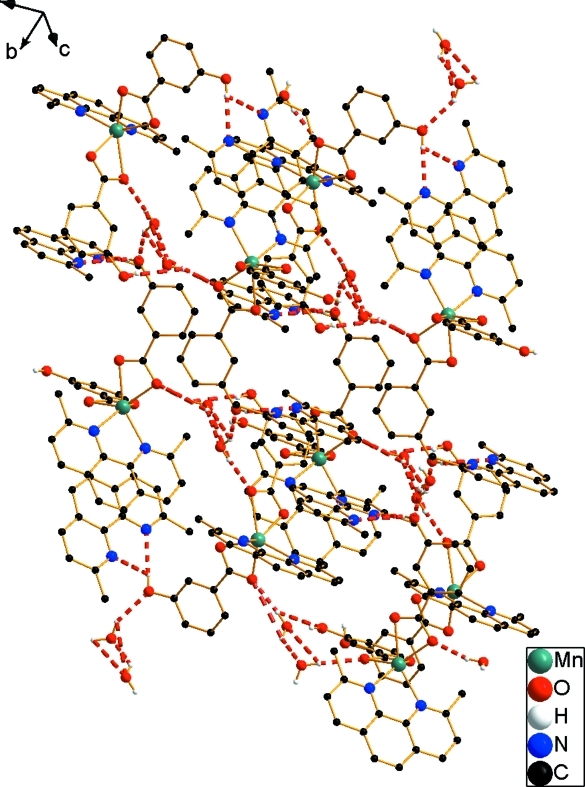
Part of the crystal structure of (I), showing the formation of hydrogen-bonded(dashed lines) and π–π stacking interactions.

## supplementary crystallographic information

### Comment

Manganese(II)-phenanthroline complexes containing benzoate anion have been extensively synthesized and reported (Xuan *et al.*, 2007; Xuan & Zhao, 2007; Wang *et al.* 2003; Zhao *et al.*, 2007; 2009). The Mn^II^ ion, in the complex molecule obtained by reaction of dmphen, sodium 3-hydroxy-benzoate and Mn(NO_3_)_2_, is six-coordinated by a bidentate dmphen ligand and two bidentate 3-hydroxybenzoate anions in a distorted octahedral environment (Xuan *et al.*, 2007). Recently, we have obtained the title compound, (I), a new Mn(II) complex following the procedure reported in the literature (Xuan *et al.*, 2007), and its structure is reported here.

The asymmetric unit of (I) (Fig. 1) is composed of a Mn-complex wherein an Mn^II^ ion is coordinated by a bidentate 2,9-dimethyl-1,10-phenanthroline (dmphen) ligand, two monodentate 3-hydroxy-benzoate anions (3-HBA) and one water molecule, one non-coordinated dmphen molecule and one bridged water molecule. The Mn atom is five-coordinated by two N atoms from dmphen ligands, three O atoms from two 3-HBAs and one water, forming a distorted triangular bipyramid geometry. The axial positions are occupied by O4 atom of 3-HBA and N1 atom of dmphen. Two 3-HBA anions act as monodentate ligands coordinated to Mn with 2.046 (3) and 2.153 (2) Å. The former is shorter than the normal Mn—O bond distance found in those similar complexes (Su & Xu, 2005). The title complex is very different from that reported in literature (Xuan *et al.*, 2007).

The crystal structure of (I) is stabilized by intramolecular O—H···O hydrogen bonds between the coordinated water and carboxylate group of 3HBA, and O—H···N hydrogen bonds between the uncoordinated water molecules and uncoordinated dmphen. The intermolecular O—H···O and O—H···N hydrogen bonds are complicated, presented by coordinated 3HBA and uncoordinated water and dmphen molecules (Table 2 and Fig. 2). In addition, π–π stacking interaction between the dmphen rings (Fig. 2) is observed with a *Cg*4 -*Cg*11^i ^separation of 3.705 (3) Å (*Cg*4 is the centroid of the C5—C8/C13—C14; *Cg*11 is the centroid of the N4/C30—C33/C42; symmetry code: (i) *x*,1/2 - *y*,1/2 + *z*).

### Experimental

The title compound was obtained unintentionally as the product of an attempted synthesis of Manganese(II)-phenanthroline complexes without uncoordinated dmphen molecule. The prepared process was similar to that of (2,9-Dimethyl-1,10-phenanthroline-*κ*^2^*N*,*N*')bis(3-hydroxybenzoato-*κ*^2^*O*,*O*')manganese(II) 2,9-dimethyl-1,10-phenanthroline dihydrate(Xuan *et al. *2007), but the molar ratio of dmphen to 3-HBA is fixed at 1:1. Yellow single crystals of (I) were obtained by slow evaporation of the filtrate over 30 days.

### Refinement

The H atoms bound to O were found *via* Fourier difference map, and refined as riding in their as-found relative positions with *U_iso_*(H) = 1.5*U_eq_*(O). Other H atoms were positioned geometrically and refined using a riding model, with fixed C—H distances of 0.93 Å (C—H) [*U*_iso_(H) = 1.2*U*_eq_(C)] and 0.96 Å (CH_3_) [*U_iso_*(H) = 1.5*U_eq_*(C)]. As for the residual electron density, the highest peak (0.920 e.Å^-3^) is 2.21 Å from H40C and the deepest hole (-0.275 e.Å^-3^) is 0.75 Å from Mn1, respectively, which indicates all atoms have been found.

### Crystal data

**Table:**

[Mn(C_7_H_5_O_3_)_2_(C_14_H_12_N_2_)(H_2_O)]·C_14_H_12_N_2_·H_2_O	*F*(000) = 1628
*M**_r_* = 781.70	*D*_x_ = 1.369 Mg m^−^^3^
Monoclinic, *P*2_1_/*c*	Mo *K*α radiation, λ = 0.71073 Å
*a* = 14.7103 (16) Å	Cell parameters from 2405 reflections
*b* = 18.578 (2) Å	θ = 2.6–20.4°
*c* = 14.4598 (16) Å	µ = 0.41 mm^−^^1^
β = 106.302 (1)°	*T* = 296 K
*V* = 3792.9 (7) Å^3^	Block, yellow
*Z* = 4	0.37 × 0.35 × 0.12 mm

### Data collection

**Table:**

Bruker SMART CCD area-detector diffractometer	7009 independent reflections
Radiation source: fine-focus sealed tube	4112 reflections with *I* > 2σ(*I*)
graphite	*R*_int_ = 0.063
φ and ω scans	θ_max_ = 25.5°, θ_min_ = 2.6°
Absorption correction: multi-scan (*SADABS*; Bruker, 1997)	*h* = −17→16
*T*_min_ = 0.864, *T*_max_ = 0.953	*k* = −22→22
22827 measured reflections	*l* = −17→17

### Refinement

**Table:**

Refinement on *F*^2^	Primary atom site location: structure-invariant direct methods
Least-squares matrix: full	Secondary atom site location: difference Fourier map
*R*[*F*^2^ > 2σ(*F*^2^)] = 0.054	Hydrogen site location: inferred from neighbouring sites
*wR*(*F*^2^) = 0.151	H-atom parameters constrained
*S* = 1.01	*w* = 1/[σ^2^(*F*_o_^2^) + (0.0679*P*)^2^ + 0.5004*P*] where *P* = (*F*_o_^2^ + 2*F*_c_^2^)/3
7009 reflections	(Δ/σ)_max_ = 0.001
502 parameters	Δρ_max_ = 0.92 e Å^−^^3^
0 restraints	Δρ_min_ = −0.28 e Å^−^^3^

### Special details

**Table:**

Geometry. All e.s.d.'s (except the e.s.d. in the dihedral angle between two l.s. planes) are estimated using the full covariance matrix. The cell e.s.d.'s are taken into account individually in the estimation of e.s.d.'s in distances, angles and torsion angles; correlations between e.s.d.'s in cell parameters are only used when they are defined by crystal symmetry. An approximate (isotropic) treatment of cell e.s.d.'s is used for estimating e.s.d.'s involving l.s. planes.
Refinement. Refinement of *F*^2^ against ALL reflections. The weighted *R*-factor *wR* and goodness of fit *S* are based on *F*^2^, conventional *R*-factors *R* are based on *F*, with *F* set to zero for negative *F*^2^. The threshold expression of *F*^2^ > σ(*F*^2^) is used only for calculating *R*-factors(gt) *etc*. and is not relevant to the choice of reflections for refinement. *R*-factors based on *F*^2^ are statistically about twice as large as those based on *F*, and *R*- factors based on ALL data will be even larger.

### Fractional atomic coordinates and isotropic or equivalent isotropic displacement parameters (Å^2^)

**Table:**

	*x*	*y*	*z*	*U*_iso_*/*U*_eq_	
Mn1	0.14385 (4)	0.32119 (3)	0.70760 (4)	0.03899 (18)	
O1	0.4872 (2)	0.20746 (15)	0.2976 (2)	0.0816 (10)	
H1W	0.4410	0.2360	0.2754	0.122*	
H2W	0.4524	0.1735	0.3025	0.122*	
O2	0.26757 (17)	0.32531 (12)	0.66050 (17)	0.0504 (6)	
H3W	0.2949	0.2859	0.6743	0.076*	
H4W	0.3059	0.3590	0.6789	0.076*	
O3	0.3326 (2)	0.19785 (13)	0.7227 (2)	0.0698 (9)	
O4	0.1872 (2)	0.21101 (13)	0.73842 (19)	0.0562 (7)	
O5	0.0852 (2)	−0.03641 (14)	0.7926 (2)	0.0727 (9)	
H5	0.0877	−0.0805	0.7948	0.109*	
O6	0.02587 (19)	0.28869 (14)	0.60321 (19)	0.0607 (7)	
O7	−0.0763 (2)	0.32079 (14)	0.68232 (18)	0.0611 (7)	
O8	−0.3901 (2)	0.24277 (17)	0.4640 (2)	0.0710 (8)	
H8	−0.4275	0.2339	0.4115	0.106*	
N1	0.1329 (2)	0.44109 (14)	0.67497 (19)	0.0402 (7)	
N2	0.13357 (19)	0.37248 (14)	0.84230 (19)	0.0393 (7)	
N3	0.3926 (2)	0.06105 (16)	0.1783 (2)	0.0514 (8)	
N4	0.3871 (2)	0.08897 (18)	0.3634 (2)	0.0519 (8)	
C1	0.1284 (3)	0.4289 (2)	0.5069 (3)	0.0676 (12)	
H1A	0.1917	0.4153	0.5080	0.101*	
H1B	0.1001	0.4557	0.4492	0.101*	
H1C	0.0916	0.3864	0.5082	0.101*	
C2	0.1313 (3)	0.47420 (19)	0.5925 (3)	0.0500 (10)	
C3	0.1338 (3)	0.5494 (2)	0.5864 (3)	0.0691 (13)	
H3	0.1359	0.5713	0.5292	0.083*	
C4	0.1331 (3)	0.5897 (2)	0.6630 (4)	0.0752 (14)	
H4	0.1346	0.6396	0.6584	0.090*	
C5	0.1303 (3)	0.5579 (2)	0.7501 (3)	0.0597 (11)	
C6	0.1251 (4)	0.5968 (2)	0.8322 (4)	0.0812 (15)	
H6	0.1250	0.6468	0.8303	0.097*	
C7	0.1204 (4)	0.5637 (3)	0.9124 (4)	0.0811 (15)	
H7	0.1159	0.5909	0.9649	0.097*	
C8	0.1222 (3)	0.4863 (2)	0.9191 (3)	0.0589 (11)	
C9	0.1168 (3)	0.4490 (3)	1.0004 (3)	0.0736 (14)	
H9	0.1120	0.4744	1.0542	0.088*	
C10	0.1184 (3)	0.3762 (3)	1.0024 (3)	0.0662 (12)	
H10	0.1132	0.3516	1.0567	0.079*	
C11	0.1281 (3)	0.3380 (2)	0.9214 (3)	0.0500 (10)	
C12	0.1318 (3)	0.2578 (2)	0.9226 (3)	0.0700 (13)	
H12A	0.0848	0.2393	0.8676	0.105*	
H12B	0.1195	0.2403	0.9804	0.105*	
H12C	0.1934	0.2422	0.9207	0.105*	
C13	0.1297 (3)	0.44604 (18)	0.8401 (2)	0.0435 (9)	
C14	0.1321 (2)	0.48206 (18)	0.7530 (2)	0.0421 (9)	
C15	0.2586 (3)	0.17416 (19)	0.7392 (2)	0.0435 (9)	
C16	0.2549 (3)	0.09441 (18)	0.7580 (2)	0.0404 (9)	
C17	0.3336 (3)	0.0513 (2)	0.7630 (3)	0.0493 (10)	
H17	0.3889	0.0715	0.7553	0.059*	
C18	0.3291 (3)	−0.0210 (2)	0.7792 (3)	0.0584 (11)	
H18	0.3820	−0.0497	0.7835	0.070*	
C19	0.2469 (3)	−0.05203 (19)	0.7893 (3)	0.0515 (10)	
H19	0.2446	−0.1012	0.8003	0.062*	
C20	0.1686 (3)	−0.00984 (19)	0.7831 (2)	0.0455 (9)	
C21	0.1730 (3)	0.06356 (18)	0.7671 (2)	0.0438 (9)	
H21	0.1200	0.0921	0.7626	0.053*	
C22	−0.0567 (3)	0.29329 (17)	0.6123 (3)	0.0435 (9)	
C23	−0.1351 (3)	0.26364 (16)	0.5298 (2)	0.0375 (8)	
C24	−0.2275 (3)	0.26573 (18)	0.5323 (3)	0.0452 (9)	
H24	−0.2423	0.2858	0.5852	0.054*	
C25	−0.2991 (3)	0.23835 (19)	0.4570 (3)	0.0489 (10)	
C26	−0.2773 (3)	0.2086 (2)	0.3786 (3)	0.0608 (11)	
H26	−0.3252	0.1905	0.3273	0.073*	
C27	−0.1842 (3)	0.2056 (2)	0.3766 (3)	0.0666 (12)	
H27	−0.1693	0.1848	0.3243	0.080*	
C28	−0.1133 (3)	0.23324 (19)	0.4515 (3)	0.0498 (10)	
H28	−0.0507	0.2315	0.4494	0.060*	
C29	0.3855 (3)	0.1785 (3)	0.4831 (3)	0.0954 (18)	
H29A	0.4504	0.1914	0.5126	0.143*	
H29B	0.3498	0.1842	0.5290	0.143*	
H29C	0.3596	0.2090	0.4285	0.143*	
C30	0.3805 (3)	0.1015 (3)	0.4508 (3)	0.0704 (13)	
C31	0.3716 (3)	0.0449 (4)	0.5139 (3)	0.097 (2)	
H31	0.3664	0.0551	0.5752	0.116*	
C32	0.3707 (4)	−0.0247 (4)	0.4829 (5)	0.104 (2)	
H32	0.3653	−0.0621	0.5238	0.125*	
C33	0.3778 (3)	−0.0405 (3)	0.3916 (4)	0.0786 (15)	
C34	0.3801 (4)	−0.1132 (3)	0.3554 (5)	0.0933 (18)	
H34	0.3764	−0.1524	0.3942	0.112*	
C35	0.3872 (4)	−0.1235 (3)	0.2675 (5)	0.0986 (18)	
H35	0.3889	−0.1705	0.2460	0.118*	
C36	0.3924 (3)	−0.0675 (2)	0.2055 (4)	0.0711 (13)	
C37	0.3979 (3)	−0.0764 (3)	0.1099 (4)	0.0848 (16)	
H37	0.3993	−0.1226	0.0858	0.102*	
C38	0.4009 (4)	−0.0201 (3)	0.0536 (4)	0.0839 (15)	
H38	0.4046	−0.0270	−0.0090	0.101*	
C39	0.3986 (3)	0.0482 (2)	0.0890 (3)	0.0652 (12)	
C40	0.4033 (4)	0.1128 (3)	0.0284 (3)	0.0960 (18)	
H40A	0.3433	0.1372	0.0120	0.144*	
H40B	0.4176	0.0978	−0.0294	0.144*	
H40C	0.4518	0.1448	0.0639	0.144*	
C41	0.3899 (3)	0.0049 (2)	0.2362 (3)	0.0514 (10)	
C42	0.3853 (3)	0.0190 (2)	0.3336 (3)	0.0527 (10)	

### Atomic displacement parameters (Å^2^)

**Table:**

	*U*^11^	*U*^22^	*U*^33^	*U*^12^	*U*^13^	*U*^23^
Mn1	0.0398 (4)	0.0366 (3)	0.0411 (3)	−0.0033 (3)	0.0122 (2)	−0.0020 (2)
O1	0.052 (2)	0.0693 (19)	0.110 (3)	−0.0079 (15)	0.0003 (18)	−0.0007 (18)
O2	0.0444 (16)	0.0410 (13)	0.0678 (16)	−0.0026 (12)	0.0188 (13)	0.0056 (12)
O3	0.0519 (19)	0.0475 (16)	0.115 (2)	−0.0035 (14)	0.0309 (18)	0.0107 (15)
O4	0.0597 (19)	0.0398 (14)	0.0804 (19)	0.0056 (14)	0.0381 (16)	0.0060 (13)
O5	0.064 (2)	0.0546 (17)	0.105 (2)	−0.0081 (16)	0.0319 (18)	0.0095 (18)
O6	0.0391 (18)	0.0731 (19)	0.0679 (18)	−0.0116 (14)	0.0116 (15)	−0.0169 (14)
O7	0.065 (2)	0.0629 (17)	0.0533 (16)	−0.0053 (15)	0.0137 (14)	−0.0156 (14)
O8	0.0403 (19)	0.086 (2)	0.078 (2)	−0.0084 (16)	0.0031 (15)	0.0093 (18)
N1	0.0388 (19)	0.0376 (16)	0.0435 (16)	−0.0010 (13)	0.0101 (14)	0.0019 (13)
N2	0.0361 (18)	0.0419 (17)	0.0400 (16)	0.0002 (14)	0.0109 (14)	0.0009 (13)
N3	0.049 (2)	0.0506 (19)	0.057 (2)	0.0096 (16)	0.0186 (16)	−0.0043 (16)
N4	0.044 (2)	0.067 (2)	0.0445 (18)	−0.0010 (17)	0.0123 (15)	−0.0038 (16)
C1	0.083 (4)	0.076 (3)	0.045 (2)	0.007 (3)	0.021 (2)	0.012 (2)
C2	0.048 (3)	0.049 (2)	0.052 (2)	0.0038 (19)	0.0115 (19)	0.0164 (18)
C3	0.072 (3)	0.056 (3)	0.074 (3)	−0.001 (2)	0.014 (3)	0.023 (2)
C4	0.081 (4)	0.036 (2)	0.101 (4)	0.001 (2)	0.014 (3)	0.015 (3)
C5	0.056 (3)	0.040 (2)	0.079 (3)	0.001 (2)	0.011 (2)	−0.010 (2)
C6	0.090 (4)	0.047 (3)	0.096 (4)	0.007 (3)	0.009 (3)	−0.017 (3)
C7	0.093 (4)	0.068 (3)	0.077 (3)	0.009 (3)	0.013 (3)	−0.037 (3)
C8	0.057 (3)	0.066 (3)	0.051 (2)	0.000 (2)	0.010 (2)	−0.018 (2)
C9	0.070 (3)	0.098 (4)	0.052 (3)	0.010 (3)	0.015 (2)	−0.025 (3)
C10	0.063 (3)	0.100 (4)	0.040 (2)	0.009 (3)	0.021 (2)	0.002 (2)
C11	0.040 (2)	0.067 (3)	0.046 (2)	0.0028 (19)	0.0174 (18)	0.0066 (19)
C12	0.083 (4)	0.063 (3)	0.074 (3)	0.008 (2)	0.040 (3)	0.027 (2)
C13	0.037 (2)	0.045 (2)	0.046 (2)	0.0013 (17)	0.0069 (17)	−0.0103 (17)
C14	0.033 (2)	0.039 (2)	0.051 (2)	−0.0007 (16)	0.0059 (17)	−0.0009 (17)
C15	0.049 (3)	0.039 (2)	0.0435 (19)	−0.0006 (19)	0.0143 (18)	0.0005 (16)
C16	0.044 (2)	0.045 (2)	0.0317 (18)	0.0005 (18)	0.0089 (16)	0.0005 (15)
C17	0.047 (3)	0.052 (2)	0.049 (2)	−0.003 (2)	0.0143 (19)	0.0015 (18)
C18	0.057 (3)	0.046 (2)	0.072 (3)	0.009 (2)	0.018 (2)	0.004 (2)
C19	0.061 (3)	0.036 (2)	0.055 (2)	0.000 (2)	0.014 (2)	−0.0004 (17)
C20	0.048 (3)	0.046 (2)	0.044 (2)	−0.010 (2)	0.0147 (18)	−0.0025 (16)
C21	0.049 (3)	0.038 (2)	0.046 (2)	0.0024 (18)	0.0153 (18)	0.0001 (16)
C22	0.049 (3)	0.0308 (18)	0.047 (2)	−0.0027 (18)	0.0085 (19)	0.0024 (16)
C23	0.039 (2)	0.0287 (18)	0.0422 (19)	−0.0001 (16)	0.0071 (17)	0.0031 (15)
C24	0.050 (3)	0.039 (2)	0.047 (2)	0.0009 (18)	0.0123 (19)	−0.0003 (16)
C25	0.039 (3)	0.042 (2)	0.060 (3)	−0.0030 (18)	0.005 (2)	0.0087 (18)
C26	0.055 (3)	0.062 (3)	0.054 (2)	−0.009 (2)	−0.005 (2)	−0.010 (2)
C27	0.068 (3)	0.073 (3)	0.057 (3)	−0.006 (2)	0.014 (2)	−0.020 (2)
C28	0.043 (3)	0.047 (2)	0.057 (2)	−0.0015 (18)	0.011 (2)	−0.0063 (18)
C29	0.066 (4)	0.150 (5)	0.070 (3)	0.001 (3)	0.017 (3)	−0.044 (3)
C30	0.047 (3)	0.111 (4)	0.052 (3)	−0.004 (3)	0.010 (2)	−0.009 (3)
C31	0.051 (3)	0.190 (7)	0.047 (3)	−0.017 (4)	0.010 (2)	0.014 (4)
C32	0.061 (4)	0.150 (6)	0.092 (5)	−0.011 (4)	0.006 (3)	0.054 (4)
C33	0.047 (3)	0.102 (4)	0.079 (3)	−0.003 (3)	0.004 (2)	0.032 (3)
C34	0.071 (4)	0.058 (3)	0.139 (5)	−0.003 (3)	0.010 (4)	0.048 (4)
C35	0.083 (4)	0.057 (3)	0.148 (6)	0.001 (3)	0.019 (4)	0.010 (4)
C36	0.047 (3)	0.051 (3)	0.109 (4)	0.002 (2)	0.011 (3)	−0.001 (3)
C37	0.068 (4)	0.078 (4)	0.108 (4)	0.004 (3)	0.025 (3)	−0.046 (3)
C38	0.075 (4)	0.099 (4)	0.080 (3)	0.009 (3)	0.024 (3)	−0.032 (3)
C39	0.057 (3)	0.077 (3)	0.067 (3)	0.016 (2)	0.025 (2)	−0.015 (2)
C40	0.112 (5)	0.114 (4)	0.078 (3)	0.036 (4)	0.053 (3)	0.016 (3)
C41	0.035 (2)	0.048 (2)	0.068 (3)	0.0042 (18)	0.007 (2)	−0.004 (2)
C42	0.035 (2)	0.060 (3)	0.060 (2)	0.0017 (19)	0.0077 (19)	0.016 (2)

### Geometric parameters (Å, °)

**Table:**

Mn1—O6	2.046 (3)	C13—C14	1.435 (5)
Mn1—O2	2.116 (2)	C15—C16	1.510 (5)
Mn1—O4	2.153 (2)	C16—C21	1.373 (5)
Mn1—N2	2.211 (3)	C16—C17	1.394 (5)
Mn1—N1	2.273 (3)	C17—C18	1.368 (5)
O1—H1W	0.8506	C17—H17	0.9300
O1—H2W	0.8278	C18—C19	1.384 (5)
O2—H3W	0.8328	C18—H18	0.9300
O2—H4W	0.8341	C19—C20	1.375 (5)
O3—C15	1.257 (4)	C19—H19	0.9300
O4—C15	1.251 (4)	C20—C21	1.388 (5)
O5—C20	1.364 (4)	C21—H21	0.9300
O5—H5	0.8200	C22—C23	1.511 (5)
O6—C22	1.261 (4)	C23—C24	1.372 (5)
O7—C22	1.238 (4)	C23—C28	1.381 (5)
O8—C25	1.375 (4)	C24—C25	1.381 (5)
O8—H8	0.8200	C24—H24	0.9300
N1—C2	1.337 (4)	C25—C26	1.377 (5)
N1—C14	1.364 (4)	C26—C27	1.379 (6)
N2—C11	1.333 (4)	C26—H26	0.9300
N2—C13	1.368 (4)	C27—C28	1.374 (5)
N3—C39	1.341 (5)	C27—H27	0.9300
N3—C41	1.344 (5)	C28—H28	0.9300
N4—C30	1.314 (5)	C29—C30	1.500 (6)
N4—C42	1.368 (5)	C29—H29A	0.9600
C1—C2	1.488 (5)	C29—H29B	0.9600
C1—H1A	0.9600	C29—H29C	0.9600
C1—H1B	0.9600	C30—C31	1.422 (7)
C1—H1C	0.9600	C31—C32	1.368 (8)
C2—C3	1.400 (5)	C31—H31	0.9300
C3—C4	1.341 (6)	C32—C33	1.384 (8)
C3—H3	0.9300	C32—H32	0.9300
C4—C5	1.402 (6)	C33—C42	1.409 (6)
C4—H4	0.9300	C33—C34	1.453 (7)
C5—C14	1.409 (5)	C34—C35	1.318 (7)
C5—C6	1.411 (6)	C34—H34	0.9300
C6—C7	1.330 (6)	C35—C36	1.389 (7)
C6—H6	0.9300	C35—H35	0.9300
C7—C8	1.440 (6)	C36—C37	1.416 (7)
C7—H7	0.9300	C36—C41	1.421 (5)
C8—C9	1.386 (6)	C37—C38	1.335 (7)
C8—C13	1.395 (5)	C37—H37	0.9300
C9—C10	1.353 (6)	C38—C39	1.371 (6)
C9—H9	0.9300	C38—H38	0.9300
C10—C11	1.411 (5)	C39—C40	1.499 (6)
C10—H10	0.9300	C40—H40A	0.9600
C11—C12	1.491 (5)	C40—H40B	0.9600
C12—H12A	0.9600	C40—H40C	0.9600
C12—H12B	0.9600	C41—C42	1.453 (5)
C12—H12C	0.9600		
			
O6—Mn1—O2	113.66 (10)	C16—C17—H17	120.2
O6—Mn1—O4	90.74 (11)	C17—C18—C19	120.9 (4)
O2—Mn1—O4	82.49 (9)	C17—C18—H18	119.6
O6—Mn1—N2	121.57 (11)	C19—C18—H18	119.6
O2—Mn1—N2	123.06 (10)	C20—C19—C18	119.8 (3)
O4—Mn1—N2	108.47 (10)	C20—C19—H19	120.1
O6—Mn1—N1	98.05 (11)	C18—C19—H19	120.1
O2—Mn1—N1	85.12 (9)	O5—C20—C19	123.2 (3)
O4—Mn1—N1	166.93 (10)	O5—C20—C21	117.2 (3)
N2—Mn1—N1	75.06 (10)	C19—C20—C21	119.6 (4)
H1W—O1—H2W	93.4	C16—C21—C20	120.6 (4)
Mn1—O2—H3W	107.0	C16—C21—H21	119.7
Mn1—O2—H4W	119.3	C20—C21—H21	119.7
H3W—O2—H4W	110.2	O7—C22—O6	124.4 (4)
C15—O4—Mn1	136.2 (2)	O7—C22—C23	119.7 (4)
C20—O5—H5	109.5	O6—C22—C23	115.9 (3)
C22—O6—Mn1	123.1 (2)	C24—C23—C28	119.5 (3)
C25—O8—H8	109.5	C24—C23—C22	120.7 (3)
C2—N1—C14	118.7 (3)	C28—C23—C22	119.7 (3)
C2—N1—Mn1	128.2 (2)	C23—C24—C25	120.7 (3)
C14—N1—Mn1	113.1 (2)	C23—C24—H24	119.7
C11—N2—C13	119.2 (3)	C25—C24—H24	119.7
C11—N2—Mn1	125.7 (2)	O8—C25—C26	122.8 (4)
C13—N2—Mn1	115.0 (2)	O8—C25—C24	117.5 (4)
C39—N3—C41	118.9 (3)	C26—C25—C24	119.7 (4)
C30—N4—C42	118.1 (4)	C25—C26—C27	119.6 (4)
C2—C1—H1A	109.5	C25—C26—H26	120.2
C2—C1—H1B	109.5	C27—C26—H26	120.2
H1A—C1—H1B	109.5	C28—C27—C26	120.5 (4)
C2—C1—H1C	109.5	C28—C27—H27	119.7
H1A—C1—H1C	109.5	C26—C27—H27	119.7
H1B—C1—H1C	109.5	C27—C28—C23	119.9 (4)
N1—C2—C3	121.4 (4)	C27—C28—H28	120.0
N1—C2—C1	118.1 (3)	C23—C28—H28	120.0
C3—C2—C1	120.5 (4)	C30—C29—H29A	109.5
C4—C3—C2	119.9 (4)	C30—C29—H29B	109.5
C4—C3—H3	120.0	H29A—C29—H29B	109.5
C2—C3—H3	120.0	C30—C29—H29C	109.5
C3—C4—C5	121.0 (4)	H29A—C29—H29C	109.5
C3—C4—H4	119.5	H29B—C29—H29C	109.5
C5—C4—H4	119.5	N4—C30—C31	122.1 (5)
C4—C5—C14	116.4 (4)	N4—C30—C29	117.3 (4)
C4—C5—C6	124.2 (4)	C31—C30—C29	120.6 (5)
C14—C5—C6	119.4 (4)	C32—C31—C30	118.8 (5)
C7—C6—C5	121.7 (4)	C32—C31—H31	120.6
C7—C6—H6	119.2	C30—C31—H31	120.6
C5—C6—H6	119.2	C31—C32—C33	121.1 (5)
C6—C7—C8	121.0 (4)	C31—C32—H32	119.4
C6—C7—H7	119.5	C33—C32—H32	119.4
C8—C7—H7	119.5	C32—C33—C42	116.1 (5)
C9—C8—C13	117.5 (4)	C32—C33—C34	123.7 (5)
C9—C8—C7	123.5 (4)	C42—C33—C34	120.1 (5)
C13—C8—C7	119.0 (4)	C35—C34—C33	119.9 (5)
C10—C9—C8	120.9 (4)	C35—C34—H34	120.1
C10—C9—H9	119.5	C33—C34—H34	120.1
C8—C9—H9	119.5	C34—C35—C36	123.2 (5)
C9—C10—C11	119.3 (4)	C34—C35—H35	118.4
C9—C10—H10	120.3	C36—C35—H35	118.4
C11—C10—H10	120.3	C35—C36—C37	124.9 (5)
N2—C11—C10	121.0 (4)	C35—C36—C41	119.8 (5)
N2—C11—C12	118.7 (3)	C37—C36—C41	115.4 (4)
C10—C11—C12	120.3 (4)	C38—C37—C36	121.7 (4)
C11—C12—H12A	109.5	C38—C37—H37	119.1
C11—C12—H12B	109.5	C36—C37—H37	119.1
H12A—C12—H12B	109.5	C37—C38—C39	119.2 (5)
C11—C12—H12C	109.5	C37—C38—H38	120.4
H12A—C12—H12C	109.5	C39—C38—H38	120.4
H12B—C12—H12C	109.5	N3—C39—C38	122.7 (4)
N2—C13—C8	122.0 (3)	N3—C39—C40	116.5 (4)
N2—C13—C14	118.4 (3)	C38—C39—C40	120.8 (4)
C8—C13—C14	119.7 (3)	C39—C40—H40A	109.5
N1—C14—C5	122.5 (3)	C39—C40—H40B	109.5
N1—C14—C13	118.3 (3)	H40A—C40—H40B	109.5
C5—C14—C13	119.2 (3)	C39—C40—H40C	109.5
O4—C15—O3	125.2 (3)	H40A—C40—H40C	109.5
O4—C15—C16	117.7 (3)	H40B—C40—H40C	109.5
O3—C15—C16	117.1 (3)	N3—C41—C36	122.2 (4)
C21—C16—C17	119.6 (3)	N3—C41—C42	118.8 (3)
C21—C16—C15	120.3 (3)	C36—C41—C42	119.0 (4)
C17—C16—C15	120.1 (3)	N4—C42—C33	123.7 (4)
C18—C17—C16	119.6 (4)	N4—C42—C41	118.3 (3)
C18—C17—H17	120.2	C33—C42—C41	117.9 (4)
			
O6—Mn1—O4—C15	122.5 (4)	Mn1—O4—C15—O3	2.9 (6)
O2—Mn1—O4—C15	8.7 (3)	Mn1—O4—C15—C16	−174.8 (2)
N2—Mn1—O4—C15	−113.7 (3)	O4—C15—C16—C21	4.4 (5)
N1—Mn1—O4—C15	−10.0 (7)	O3—C15—C16—C21	−173.6 (3)
O2—Mn1—O6—C22	−168.8 (3)	O4—C15—C16—C17	−177.8 (3)
O4—Mn1—O6—C22	109.1 (3)	O3—C15—C16—C17	4.3 (5)
N2—Mn1—O6—C22	−3.2 (3)	C21—C16—C17—C18	−1.6 (5)
N1—Mn1—O6—C22	−80.6 (3)	C15—C16—C17—C18	−179.4 (3)
O6—Mn1—N1—C2	−58.4 (3)	C16—C17—C18—C19	1.0 (6)
O2—Mn1—N1—C2	54.9 (3)	C17—C18—C19—C20	−0.1 (6)
O4—Mn1—N1—C2	73.5 (6)	C18—C19—C20—O5	−179.7 (3)
N2—Mn1—N1—C2	−179.0 (3)	C18—C19—C20—C21	−0.3 (5)
O6—Mn1—N1—C14	124.7 (2)	C17—C16—C21—C20	1.2 (5)
O2—Mn1—N1—C14	−122.1 (2)	C15—C16—C21—C20	179.1 (3)
O4—Mn1—N1—C14	−103.5 (5)	O5—C20—C21—C16	179.2 (3)
N2—Mn1—N1—C14	4.0 (2)	C19—C20—C21—C16	−0.3 (5)
O6—Mn1—N2—C11	85.3 (3)	Mn1—O6—C22—O7	3.6 (5)
O2—Mn1—N2—C11	−110.6 (3)	Mn1—O6—C22—C23	−177.4 (2)
O4—Mn1—N2—C11	−17.5 (3)	O7—C22—C23—C24	−1.1 (5)
N1—Mn1—N2—C11	175.6 (3)	O6—C22—C23—C24	179.8 (3)
O6—Mn1—N2—C13	−92.6 (3)	O7—C22—C23—C28	179.7 (3)
O2—Mn1—N2—C13	71.6 (3)	O6—C22—C23—C28	0.7 (5)
O4—Mn1—N2—C13	164.6 (2)	C28—C23—C24—C25	−0.6 (5)
N1—Mn1—N2—C13	−2.2 (2)	C22—C23—C24—C25	−179.8 (3)
C14—N1—C2—C3	3.3 (6)	C23—C24—C25—O8	−179.5 (3)
Mn1—N1—C2—C3	−173.5 (3)	C23—C24—C25—C26	0.1 (5)
C14—N1—C2—C1	−177.7 (3)	O8—C25—C26—C27	−179.7 (4)
Mn1—N1—C2—C1	5.5 (5)	C24—C25—C26—C27	0.8 (6)
N1—C2—C3—C4	−3.3 (7)	C25—C26—C27—C28	−1.1 (6)
C1—C2—C3—C4	177.7 (4)	C26—C27—C28—C23	0.6 (6)
C2—C3—C4—C5	0.2 (7)	C24—C23—C28—C27	0.3 (5)
C3—C4—C5—C14	2.5 (7)	C22—C23—C28—C27	179.4 (3)
C3—C4—C5—C6	−177.0 (5)	C42—N4—C30—C31	−0.1 (6)
C4—C5—C6—C7	178.4 (5)	C42—N4—C30—C29	178.2 (4)
C14—C5—C6—C7	−1.0 (7)	N4—C30—C31—C32	0.6 (7)
C5—C6—C7—C8	1.2 (8)	C29—C30—C31—C32	−177.6 (5)
C6—C7—C8—C9	−179.5 (5)	C30—C31—C32—C33	−0.4 (8)
C6—C7—C8—C13	0.4 (7)	C31—C32—C33—C42	−0.3 (8)
C13—C8—C9—C10	−0.2 (7)	C31—C32—C33—C34	178.2 (5)
C7—C8—C9—C10	179.7 (4)	C32—C33—C34—C35	−179.8 (6)
C8—C9—C10—C11	1.6 (7)	C42—C33—C34—C35	−1.3 (8)
C13—N2—C11—C10	0.0 (5)	C33—C34—C35—C36	−0.4 (9)
Mn1—N2—C11—C10	−177.7 (3)	C34—C35—C36—C37	−178.4 (5)
C13—N2—C11—C12	179.6 (3)	C34—C35—C36—C41	−0.1 (8)
Mn1—N2—C11—C12	1.8 (5)	C35—C36—C37—C38	178.8 (5)
C9—C10—C11—N2	−1.5 (6)	C41—C36—C37—C38	0.4 (7)
C9—C10—C11—C12	178.9 (4)	C36—C37—C38—C39	−0.1 (8)
C11—N2—C13—C8	1.4 (5)	C41—N3—C39—C38	0.9 (7)
Mn1—N2—C13—C8	179.4 (3)	C41—N3—C39—C40	−178.9 (4)
C11—N2—C13—C14	−177.8 (3)	C37—C38—C39—N3	−0.6 (8)
Mn1—N2—C13—C14	0.2 (4)	C37—C38—C39—C40	179.2 (5)
C9—C8—C13—N2	−1.4 (6)	C39—N3—C41—C36	−0.5 (6)
C7—C8—C13—N2	178.8 (4)	C39—N3—C41—C42	178.7 (4)
C9—C8—C13—C14	177.8 (4)	C35—C36—C41—N3	−178.5 (4)
C7—C8—C13—C14	−2.1 (6)	C37—C36—C41—N3	−0.1 (6)
C2—N1—C14—C5	−0.4 (5)	C35—C36—C41—C42	2.2 (6)
Mn1—N1—C14—C5	176.9 (3)	C37—C36—C41—C42	−179.3 (4)
C2—N1—C14—C13	177.3 (3)	C30—N4—C42—C33	−0.7 (6)
Mn1—N1—C14—C13	−5.4 (4)	C30—N4—C42—C41	178.3 (4)
C4—C5—C14—N1	−2.5 (6)	C32—C33—C42—N4	0.9 (6)
C6—C5—C14—N1	177.0 (4)	C34—C33—C42—N4	−177.7 (4)
C4—C5—C14—C13	179.8 (4)	C32—C33—C42—C41	−178.1 (4)
C6—C5—C14—C13	−0.7 (6)	C34—C33—C42—C41	3.4 (6)
N2—C13—C14—N1	3.6 (5)	N3—C41—C42—N4	−2.1 (5)
C8—C13—C14—N1	−175.6 (3)	C36—C41—C42—N4	177.2 (3)
N2—C13—C14—C5	−178.6 (3)	N3—C41—C42—C33	176.9 (4)
C8—C13—C14—C5	2.2 (5)	C36—C41—C42—C33	−3.8 (6)

### Hydrogen-bond geometry (Å, °)

**Table:**

*D*—H···*A*	*D*—H	H···*A*	*D*···*A*	*D*—H···*A*
O8—H8···O1^i^	0.82	1.83	2.653 (4)	176
O5—H5···O7^ii^	0.82	1.88	2.686 (4)	168
O2—H4W···N3^iii^	0.83	1.96	2.764 (4)	162
O2—H3W···O3	0.83	1.80	2.617 (3)	165
O1—H2W···N4	0.83	2.15	2.951 (4)	161
O1—H1W···O3^iv^	0.85	1.99	2.839 (4)	180

Symmetry codes: (i) *x*−1, *y*, *z*; (ii) −*x*, *y*−1/2, −*z*+3/2; (iii) *x*, −*y*+1/2, *z*+1/2; (iv) *x*, −*y*+1/2, *z*−1/2.
